# Mindfulness meditation modulates reward prediction errors in a passive conditioning task

**DOI:** 10.3389/fpsyg.2015.00090

**Published:** 2015-02-12

**Authors:** Ulrich Kirk, P. Read Montague

**Affiliations:** ^1^Department of Psychology, University of Southern DenmarkOdense, Denmark; ^2^Wellcome Trust Centre for Neuroimaging, University College LondonLondon, UK; ^3^Human Neuroimaging Laboratory, Virginia Tech Carilion Research Institute, Virginia Polytechnic Institute and State UniversityRoanoke, VA, USA; ^4^Department of Physics, Virginia Polytechnic Institute and State UniversityBlacksburg, VA, USA

**Keywords:** fMRI BOLD, mindfulness, reward processing, conditioning, classical, interoceptive awareness

## Abstract

Reinforcement learning models have demonstrated that phasic activity of dopamine neurons during reward expectation encodes information about the predictability of reward and cues that predict reward. Self-control strategies such as those practiced in mindfulness-based approaches is claimed to reduce negative and positive reactions to stimuli suggesting the hypothesis that such training may influence basic reward processing. Using a passive conditioning task and fMRI in a group of experienced mindfulness meditators and age-matched controls, we tested the hypothesis that mindfulness meditation influence reward and reward prediction error (PE) signals. We found diminished positive and negative PE-related blood-oxygen level-dependent (BOLD) responses in the putamen in meditators compared with controls. In the meditator group this decrease in striatal BOLD responses to reward PE was paralleled by increased activity in posterior insula, a primary interoceptive region. Critically, responses in the putamen during early trials of the conditioning procedure (run 1) were elevated in both meditators and controls. Overall, these results provide evidence that experienced mindfulness meditators are able to attenuate reward prediction signals to valenced stimuli, which may be related to interoceptive processes encoded in the posterior insula.

## INTRODUCTION

The inability of individuals to use self-control gives rise to a range of adverse health consequences, conferring great personal and societal costs (Mokdad et al., 2004; Schroeder, 2007). Only recently have studies in cognitive neuroscience begun to address the issue of the underlying neural mechanisms involved in self-control (Hare et al., 2009; Kirk et al., 2011a; Hutcherson et al., 2012). The clinical and social importance of self-control (Moffitt et al., 2011), as well as the possibility that sustained self-control techniques such as those practiced in mindfulness-based approaches, may be acquired and impact distinct domains of human reward processing and decision-making, provides urgency and relevance to this nascent field of study.

There is reason to expect a fundamental involvement of the dopaminergic pathways in an inability to exert self-control. The neurotransmitter system most strongly implicated in value-based decision-making is dopamine, and the mesolimbic dopaminergic system, the key substrate of the brain’s reward system, has been implicated in conditions with learning and decision-making deficits such as addiction (Montague, 2007). Despite advances in understanding how the brain assigns value to the expectancy and predictability of rewards (Montague et al., 1996; Schultz et al., 1997), a purported interaction between mindfulness-based approaches and valuation of reward prediction signals remains poorly understood.

One behavioral therapy that seems to provide systematic training in self-control and emotion management is mindfulness (Vago and Silbersweig, 2012). Mindfulness teaches practitioners to step back from emotions by enabling practitioners to experience “space between one’s perception and response” (Shapiro et al., 2006), Indeed, recent work have shown using a cross-sectional design that long-term mindfulness practice impacts an individual’s ability to regulate anticipatory responses towards monetary gains or losses in a financial incentive task by a dampening of the mesolimbic dopaminergic system, specifically the caudate nucleus (Kirk et al., 2014b). Recent studies have shown that mindfulness training enhances interoceptive awareness through practices such as breath monitoring (Farb et al., 2007, 2013b; Daubenmier et al., 2013; Kirk et al., 2014a). These reports link nicely up to the conceptual content of the actual mindfulness exercises. For example in one canonical exercise mindfulness practitioners are instructed to attend to physical sensations of breathing in a non-evaluative manner and to notice the occurrence of thoughts, emotions, sounds, and other stimuli as they arise. Once practitioners become distracted or lost in thought, attention is directed back to the breath. In a recent study involving a randomized design with 8 weeks of mindfulness training on an initially naïve subject cohort, we studied the impact of mindfulness on behavior and neural responses during value computation using both a primary and a secondary reward task (Kirk et al., 2014a). In contrast to an active control group, the mindfulness group decoupled activity in the ventromedial prefrontal cortex (vmPFC) during value computation, reflected by a suppression of vmPFC activity in this group. Instead, the mindfulness group recruited value signals that scaled linearly with preference in the left mid/anterior insula. This brain region has been proposed to play a role in attending to self-reference in the present moment (Farb et al., 2007, 2010, 2013b) and attending to internal bodily states (Critchley et al., 2004; Craig, 2009) as well as the homeostatic state of the body (Craig, 2003; Seth et al., 2011). It has recently been proposed that interoceptive functioning contributes to value-based decision-making (Gu and FitzGerald, 2014). These findings argue for the possibility that in the Kirk et al. (2014a) study the mindfulness group were able to maintain interoceptive awareness (e.g., attending to internal bodily states and presumably breath monitoring), by integrating such signals during value computation. The results further showed a decoupling between the posterior insula, and the vmPFC valuation systems in the mindfulness group in the context of both the primary and secondary reward tasks, suggesting a specific interaction mediated by mindfulness training between interoceptive networks and value computation systems. In related studies by Farb et al. (2007, 2013b), changes associated with interoceptive awareness were reported in relation to mindfulness training. Specifically, increased activation of the somatosensory and posterior insula was found in a mindfulness group when attending to present-moment experience. Farb et al. (2007) showed that present-moment experience, as a function of mindfulness training, was associated with increased activation of viscerosomatic regions, including the insula and somatosensory cortex, and reduced activation of the medial prefrontal cortex encompassing the vmPFC. In addition, mindfulness practice time has been positively correlated with activation in the posterior insula during respiratory awareness tasks (Farb et al., 2013a,b), but not all studies have found such a relationship (Kirk et al., 2011b, 2014a,b). Based on this evidence we propose that the elevated activity in posterior insula, presumably reflecting interoceptive awareness of respiration or breath monitoring, may be one basis by which mindfulness training promotes adaptive decision-making.

In the current study we set out to study if long-term meditators were able to exert self-control by focusing on interoceptive awareness while exposed to primary reward (fruit juice) and reward predicting cues (yellow circle) in a conditioning paradigm. If long-term meditators were able to control their reward response, then BOLD responses measured with fMRI in the mesolimbic dopaminergic system should exhibit a modulation compared with a control group. Specifically, based on previous reports we had *a priori* hypotheses targeting bilateral putamen (McClure et al., 2003). In addition we expected that the influence of interoceptive awareness on prediction error (PE) signaling would be encoded by elevated activity in posterior insula in the meditator group.

**FIGURE 1:**
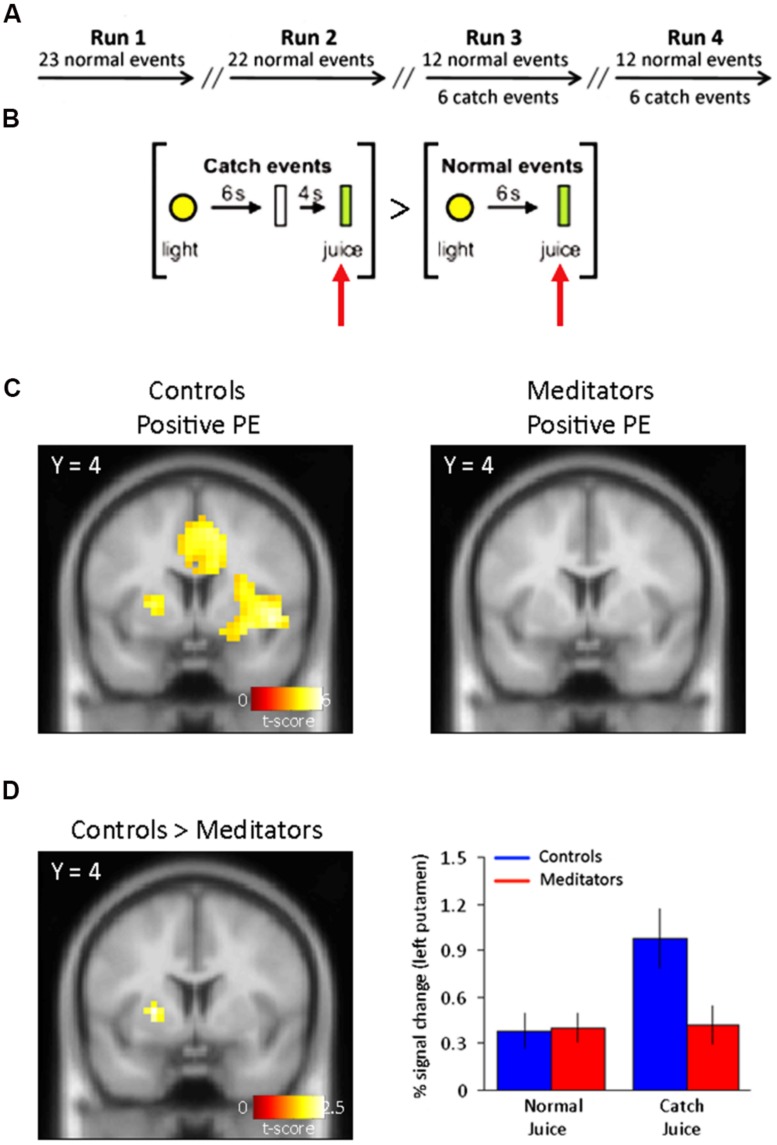
**Task outline and positive PE signals.** **(A)** Outline of the conditioning task. fMRI scanning consisted of four separate sessions/runs. Catch events were interspersed among the normal events in run 3 and run 4. Run 1 and run 2 consisted on normal (training) trials only. **(B)** Normal events consisted of a yellow light (1 s) predicting the oral delivery of fruit juice (0.8 ml) 6 s later. Catch events designed to capture a positive reward PE consisted of presentation of the light cue (1 s) and juice delivery 10 s later at an unexpected time. The specific contrast designed to capture the positive PE was: [Juice delivered (unexpected) > Juice delivered (expected)]. **(C)** Left panel, positive PE for controls display activity in bilateral putamen. Right panel, positive PEs in meditators did not yield significant voxels in the putamen (see **Table 1** for complete list of activations). **(D)** Left panel, group differences to positive reward PEs show SVC-corrected activity in left putamen in controls. Right panel, parameter estimates for the significant voxels in left putamen show an increase in the BOLD signal at times when juice was not expected but delivered. Controls shown in blue and meditators in red. Error bars indicate SE.

To test our experimental predictions, we recruited 58 participants (30 controls and 28 Buddhist meditators) and used a classical conditioning procedure that has been previously used to study reward prediction and cues that predict reward (McClure et al., 2003; Salas et al., 2010). Specifically, participants were presented with a yellow light cue (1 s), which predicted the time of reward delivery (juice). Training consisted of reliably pairing a light cue (presented centrally in participants’ visual field) with oral juice delivery 6 s later. After 45 of such normal events divided into two runs (run 1 = 23 events; run 2 = 22 events), 6 catch events were introduced in run 3 and another 6 catch events in run 4, randomly spaced in between 12 normal events. In the catch events, juice delivery was delayed 4 s beyond the time expected from training events (**Figure 1A**). Throughout the conditioning procedure juice delivery remained constant (0.8 ml). This conditioning procedure is known to generate two types of PEs during catch events: (1) no juice delivery at expected times generates a negative PE (less than expected), and (2) juice delivery at an unexpected times generates a positive PE (more than expected).

## NEUROIMAGING RESULTS

### BOLD SIGNALS REFLECT PREDICTION ERRORS IN CONTROLS

We first focused our analysis on neural effects in catch vs. normal events in run 3 and 4. In the control group, we found significant activity for unpredicted juice delivery compared with predicted delivery, [Juice delivered (unexpected) > Juice delivered (expected)] (**Figure 1B**) corresponding to a positive PE in bilateral putamen (Right: 24 4 8; *z* = 4.16. Left: –24 4 4; *z* = 5.48), significant at *p* < 0.05 false discovery rate (FDR) corrected (**Figure 1C**, left panel). No regions showed significantly greater responses to predicted delivery compared to unpredicted juice delivery [Juice delivered (expected) > Juice delivered (unexpected)].

**FIGURE 2:**
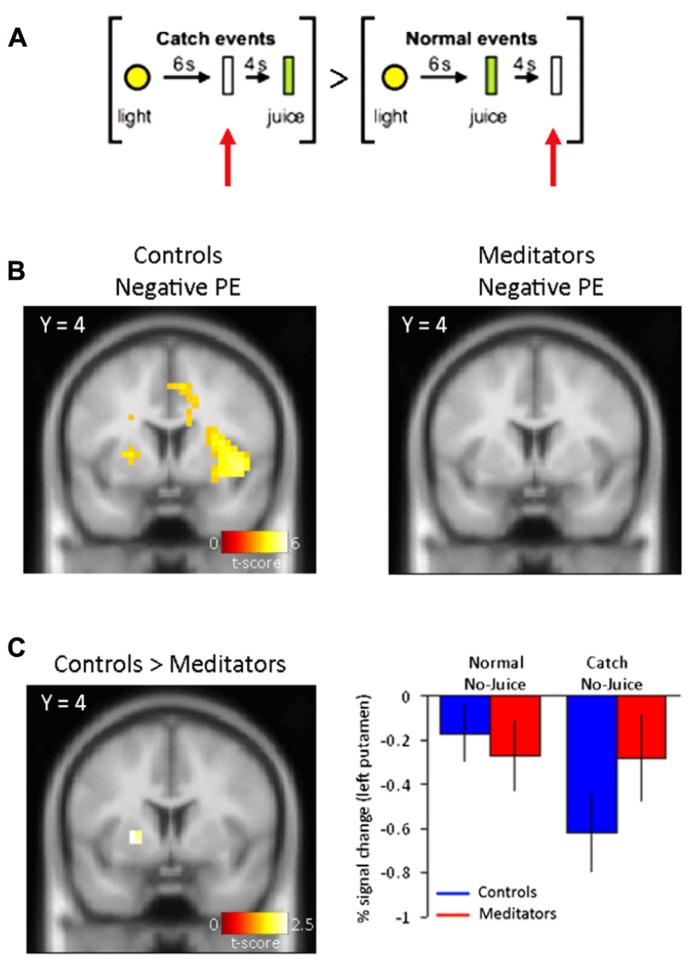
**Negative PE signals.** **(A)** Normal events consisted of a yellow light (1 s) and no juice delivery 10 s following the light cue. Catch events designed to capture a negative reward PE consisted of presentation of the light cue (1 s) and no juice delivery at the expected time at 6 s following the light cue. The specific contrast designed to capture the negative PE was: [Juice not delivered (expected) > Juice not delivered (unexpected)]. **(B)** Left panel, negative PEs for controls display activity in bilateral putamen. Right panel, negative PEs in meditators did not yield significant voxels (see **Table 1** for complete list of activations). **(C)** Left panel, group differences to negative reward PEs show activity in left putamen in controls. Right panel, parameter estimates for the significant voxels in left putamen display a drop in the BOLD signal at times when juice was expected but not delivered. Controls are shown in blue and meditators in red. Error bars indicate SE.

In catch trials where juice was expected but not delivered compared to normal trials in which juice was not expected and not delivered [Juice not delivered (expected) > Juice not delivered (unexpected)](**Figure 2A**), we found significant activity, corresponding to a negative PE, in bilateral putamen (Right: 24 4 8; z = 3.54. Left: –24 4 4; z = 4.76) significant at *p* < 0.05 FDR-corrected (**Figure 2B**, left panel). No brain regions demonstrated significantly greater changes in brain responses during normal non-delivery minus catch events [Juice not delivered (unexpected) > Juice not delivered (expected)].

**Table 1:** Summary of brain regions in both groups displaying prediction error-related contrasts.

Region	Laterality	x	y	z	*z*-score
**Control catch juice Control normal juice**
Putamen	R	24	4	8	4.16
Putamen	L	-24	4	4	5.48
Cingulate gyrus	R∖L	4	0	40	4.85
Thalamus	R∖L	4	-16	8	4.77
**Control catch no-juice Control normal no-juice**
Putamen	R	24	4	8	3.54
Cingulate gyrus	L	-24	4	4	4.76
Thalamus	R∖L	4	2	38	3.71
**Meditator catch juice Meditator normal juice**
Thalamus	R/L	4	-4	8	3.87
**Meditator catch no-juice Meditator normal no-juice**
None					

*Activations thresholded at *p* < 0.05, FDR. Extent threshold > 5 voxels.*

### REDUCED BOLD RESPONSES TO PREDICTION ERRORS IN MEDITATORS

In the meditator group, we did not observe significant activity with PE signals either positive [Juice delivered (unexpected) > Juice delivered (expected)], or negative [Juice not delivered (expected) > Juice not delivered (unexpected)] in the striatum even at *p* < 0.001, uncorrected (**Figure 1C**, right panel and **Figure 2B**, right panel). Only when dropping the threshold substantially did we observe PE-related signals in bilateral putamen (*p* < 0.05, uncorrected). No regions showed significantly greater responses to predicted delivery compared to unpredicted juice delivery [Juice delivered (expected) > Juice delivered (unexpected)]. And finally, no brain regions demonstrated significantly greater changes in brain responses during normal non-delivery minus catch events [Juice not delivered (unexpected) > Juice not delivered (expected)].

In a whole brain analysis, we show all regions that survived whole brain correction at *p* < 0.05 (FDR-corrected) in both groups for PE-related contrasts (**Table 1**).

### GROUP DIFFERENCES IN PREDICTION ERROR-RELATED ACTIVITY IN PUTAMEN

In a direct statistical comparison between positive PEs in the control and meditator group (Controls > Meditators), we found that controls showed significantly greater positive PE-related activity in the left putamen (-24 4 4; *z* = 2.42), but not the right putamen, compared with meditators (**Figure 1D**, left panel). This analysis was significant at *p* < 0.05, uncorrected, and at *p* < 0.05 FDR-corrected threshold after small volume correction (SVC; Worsley et al., 1996) of the *a priori* defined putamen. Parameter estimates of the direct comparison between controls and meditators extracted from the left putamen are shown (**Figure 1D**, right panel). The opposite contrast (Meditators > Controls) did not result in significant activity at *p* < 0.001, uncorrected.

Furthermore, in a comparison between negative PE-related activity in the control and meditator group (Controls > Meditators), we found significant activity in the left putamen (-22 4 2; *z* = 2.28). This contrast was significant at *p* < 0.05, uncorrected, and at *p* < 0.05 FDR-corrected threshold after SVC of the *a priori* defined putamen (**Figure 2C**, left panel). Parameter estimates of the direct comparison between controls and meditators extracted from the left putamen are shown (**Figure 2C**, right panel). The opposite contrast for negative PE-related activity (Meditators > Controls) did not result in significant activity at *p* < 0.001, uncorrected.

### INCREASED POSTERIOR INSULA ACTIVITY PARALLEL DECREASED PREDICTION ERRORS IN MEDITATORS

**FIGURE 3:**
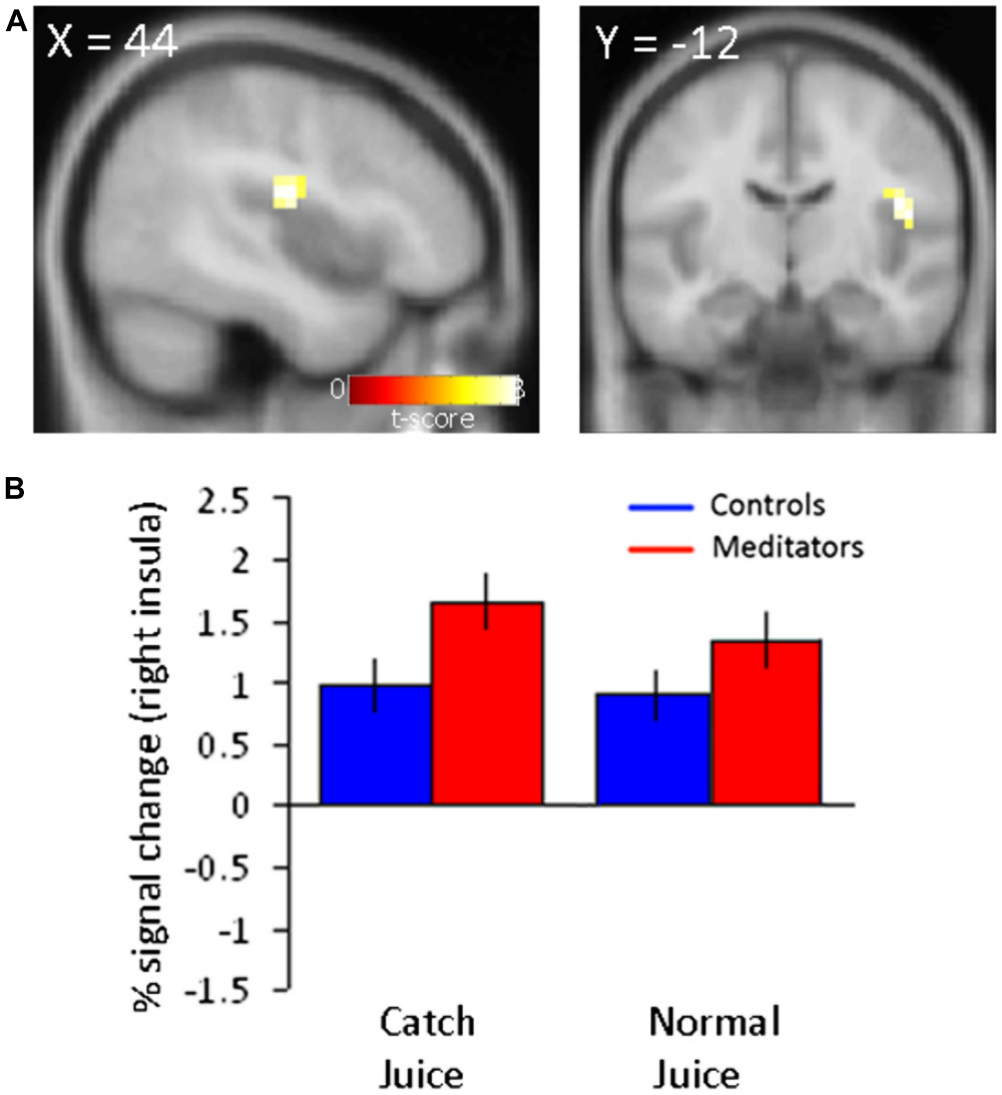
**Increased neural activity in right posterior insula to juice delivery in meditators.** **(A)** Main effect of juice delivery collapsed across modality (normal/catch) in (Meditators > Controls) resulted in activity in the right insula (44 -12 20; *z* = 3.35; *p* < 0.001, unc.). **(B)** Parameter estimates extracted from the right posterior insula region are displayed for both groups and separated into trial types corresponding to averaged responses from run 3 and 4. Error bars indicate SE.

The reduced reliance on PEs in meditators expressed as reduced BOLD responses in the putamen, suggests a process whereby mindfulness meditation enables the brain to diminish the impact of PEs on behavior. If such a process is indeed at play in this group of meditators, there should also be a corresponding increase in brain regions that mediate the implementation of reduced PE signaling. To determine which brain regions may enable the effects of reduced PEs, we conducted an exploratory analysis to localize potential brain areas where activation to juice reward (independent of modality, i.e., normal or catch trials) was greater for meditators compared with controls. We focused on juice delivery independent of modality because our previous analyses did not identify differences between catch and normal juice delivery in meditators. We computed the main effect at juice delivery time in run 3 and run 4 (Meditators > Controls). This analysis revealed activity in the right posterior insula at *p* < 0.001, uncorrected (**Figure 3A**). Parameter estimates extracted from this region demonstrated that the meditator group showed elevated activity in the posterior insula compared to controls at juice delivery both during normal juice delivery and during catch juice trials in run 3 and 4 (**Figure 3B**).

### REGIONS OF INTEREST ANALYSIS DURING TRAINING (RUN 1): PUTAMEN

**FIGURE 4:**
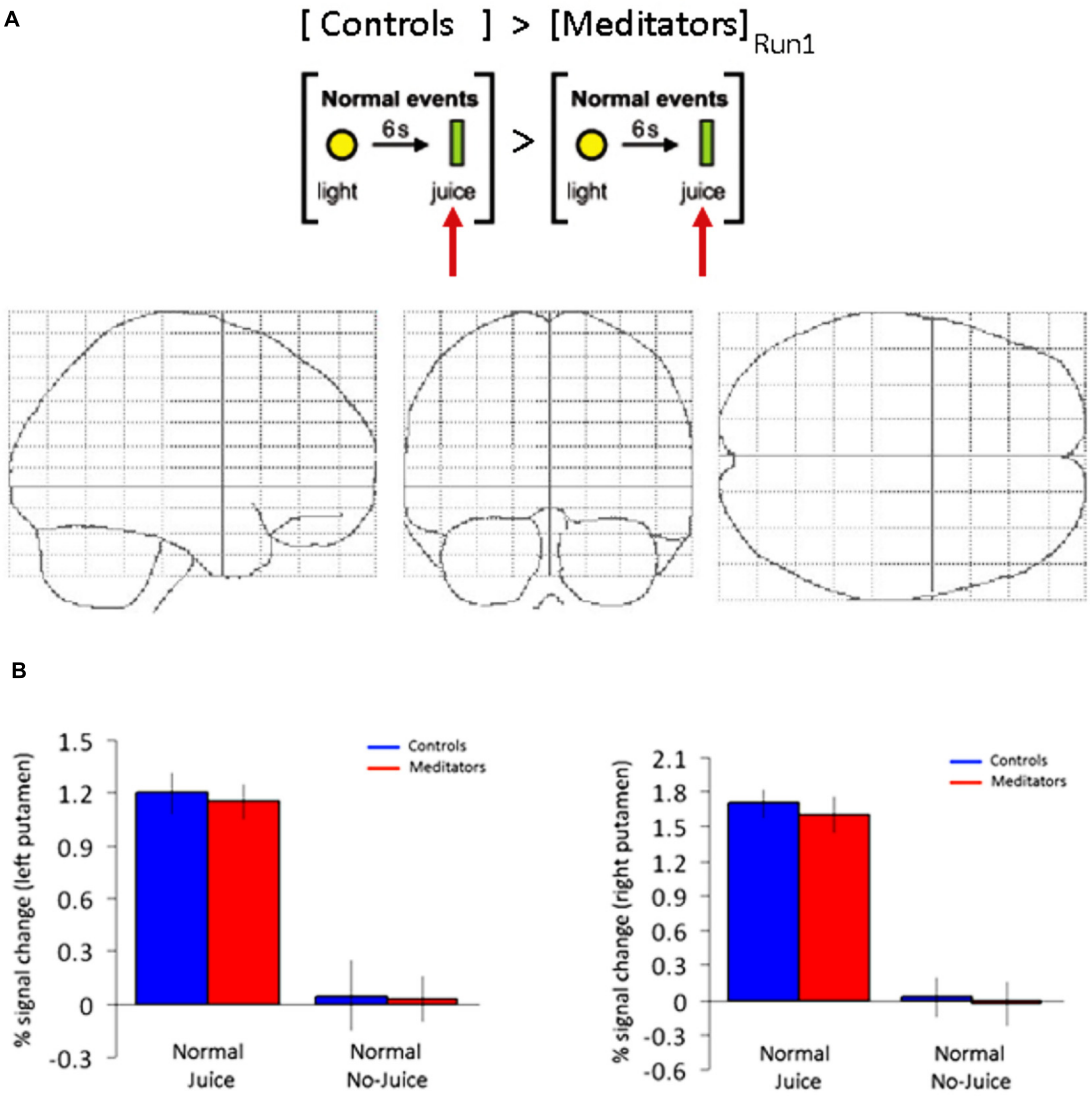
**ROI putamen: no group-specific differences during training events (run 1).** **(A)** Main effect of juice delivery (Controls > Meditators) averaged across run 1 display no differential activity in a whole brain analysis at *p* < 0.001, uncorrected. **(B)** ROI in left and right putamen. Beta estimates display significant activity in both groups, but non-differential reward activity across groups at the time of juice delivery during run 1 as displayed in **(A)**. Error bars indicate SE.

Next we tested if the differences in reward processing in the striatum was induced by the conditioning procedure or alternatively was task-independent and a pre-existing difference between the meditators and controls. We modeled the (unexpected) juice delivery time during run 1 in both groups. The contrast was computed at juice delivery 6 s after cue vs. baseline 5 s prior to cue (23 events). We found significant activity at the FDR-corrected level in bilateral putamen in both controls (Left: -24 4 12; *z* = 4.58. Right: 24 4 4; *z* = 4.45) and meditators (Left: -23 4 6; *z* = 4.58. Right: 28 0 4; *z* = 4.51). The beta estimates in the putamen did not differentiate between the two groups during unexpected juice delivery in run 1 (**Figure 4B**). In a direct comparison between the two groups at the time of (unexpected) juice delivery in run 1 we did not observe any differences between groups at *p* < 0.001, uncorrected (**Figure 4A**). These results demonstrate that mediators and controls exhibit non-differential striatal responses to unexpected juice delivery in run 1.

### REGIONS OF INTEREST ANALYSIS: VISUAL CORTEX

**FIGURE 5:**
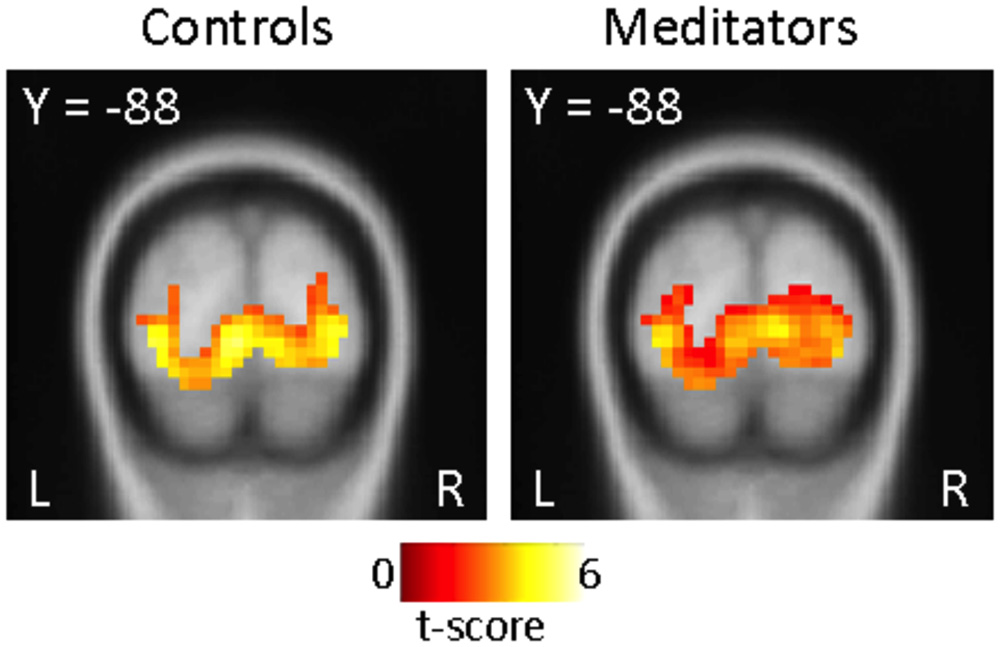
**ROI visual cortex: no group-specific differences during cue onset (run 3 and 4).** Significant activity in visual cortical areas related to light cue onset in run 3 and 4 in both controls (left panel) and meditators (right panel) displayed at *p* < 0.001, uncorrected. Direct contrast between groups (Controls > Meditators) at cue onset display no significant differences in activity at *p* < 0.001, uncorrected. The opposite contrast (Meditators > Controls) did not results in significant activity at *p* < 0.001, uncorrected.

Furthermore, we tested if differences in visual cortical areas in run 3 and 4 would account for differences in PE signaling between groups. We observed activity in primary visual cortex to the light cue in both groups (**Figure 5**), and no significant differences were found in a direct comparison (Figure not shown). These findings demonstrate that the participants in both groups were processing the visual components of the task non-differentially, and argues against the possibility that the meditator group were simply disengaged from the task.

## DISCUSSION

In this study we found that long-term meditators were better able to dampen the impact of reward PEs encoded in the ventral striatum. The findings suggest that elevated activity in posterior insula, presumably reflecting interoceptive awareness of respiration or breath monitoring, may be one basis through which mindfulness training exerts a behavioral impact on value-based decision-making. Alas, since the current design was cross-sectional, further longitudinal designs are required to complement these findings before a firm link between mindfulness training and striatal PE-related activity may be made.

Recent work using cognitive strategies such as emotion regulation have observed dampening of the striatum to reward expectancy of secondary reward (Delgado et al., 2008) and in anterior insula to fictive reward predicting errors (Gu et al., 2014). Different from emotion regulation (Ochsner and Gross, 2005), mindfulness takes an objective or outside perspective on one’s experiences, rather than changing the content (distraction) or context (reframing) of those experiences. It seems plausible that with a mindful stance the practitioner adopts emotion regulation using different mechanisms; specifically mindfulness training seems to enhance interoceptive awareness through practices such as breath monitoring (Farb et al., 2007, 2013b; Daubenmier et al., 2013; Kirk et al., 2014a).

Mindfulness has informed a number of psychoeducational interventions where popular versions include Mindfulness Based Stress Reduction (MBSR; Kabat-Zinn, 1990) and Mindfulness Based Cognitive Therapy (MBCT; Segal et al., 2001). Mindfulness as taught in such interventions differs from recent formulations of emotional regulation in three main ways. First, rather than averting attention away from difficult thoughts, mindfulness training has participants notice or accept such thoughts without reacting to them. The idea is that simply registering and staying with the moment-to-moment attention of the particular thought or feeling is a non-habitual way of responding (as oppose to reacting) to a given thought or feeling. Second, mindfulness is non-judgmental, which limits the elaborative processes mediating the appraisal of emotionally relevant experience. Third, the meta-cognitive awareness cultivated through mindfulness training promotes an experience of negative thoughts or feelings as simply mental events, rather than as real events that are self-relevant and to be acted upon. This form of training is hypothesized to have long-lasting and potent effects on economic decision-making (Kirk et al., 2014b) and cooperative exchange (Kirk et al., 2011b), however, this remains to be tested empirically and modeled quantitatively in longitudinal and experimental studies. Indeed, one limitation of the current results is that we used a cross-sectional rather than longitudinal design. Hence, it was not possible to compare the behavior of the subjects before and after they started practicing mindfulness. Without this information, we cannot determine whether the meditators actually acquired a different behavioral profile through meditation experience.

A separate line of research may offer an interpretation to the current results. Evidence suggests that mindfulness may impact mind wandering. Specifically, people who report higher trait mindfulness demonstrate less mind wandering (Mrazek et al., 2012). In addition, it has recently been found that only 2 weeks of mindfulness training decrease mind wandering and improves GRE scores (Mrazek et al., 2013). One interpretation of the current results may be that focusing on interoceptive awareness in the present moment may decrease mind wandering and thus reduce the tendency to focus on the future or past. This may have the consequence that any future state would have the same value as the current state for mindfulness practitioners, which would result is flat value functions, i.e., PE signals in this group. Indeed this interpretation seems in line with recent work suggesting that mindfulness training may accomplish reduction is bias effects by drawing the practitioners focus away from the future and past in the context of sunk cost economic decisions (Hafenbrack et al., 2013).

In summary, we found that experienced meditators exhibited reduced neural responses to reward PEs. The data suggest that the reward system in this group is not by default attenuated in that we observed significant and non-differential activity in the ventral striatum during reward delivery (juice) during the training trials (run 1) of the conditioning procedure. This argues against the simple interpretation that the meditators exhibit trait-specific reward sensitivity. Our results rather argue that interoceptive awareness processed in the posterior insula decreases the impact of the reward system. It is noteworthy that meditators were not given instructions to meditate during the scanning sessions. Participants in both groups were instructed to focus on the light cue and swallow juice as it was delivered, and no reference was made to the cue/juice pairings. Our cross-sectional design and thus preliminary data suggest that mindfulness may be an intrinsic mechanism for altered reward processing with potential clinical benefits, which would be interesting to investigate in future studies. Despite advances in understanding how the brain assigns value to the expectancy of rewards, it is poorly understood how to control or regulate hyper-valuation of reward prediction signals. The current results suggest that meditators may systematically train strategies that allow regulation and deploy self-control to primary reward predicting stimuli.

## MATERIALS AND METHODS

### SUBJECTS

Fifty eight subjects participated in the study. Subjects were recruited in two groups. One group (*n* = 30) consisted of controls. The second group (*n* = 28) consisted of expert meditators. The expert group was selected primarily from a southwestern Zen center in the US and were recruited based on the criterion of maintaining a regular mindfulness-integrated meditation practice (minimum three sessions of 20 min per week). In addition, all participants in the meditation group had completed at least one meditation retreat of min 3 days duration. Both groups maintained a normal secular lifestyle. We did not collect data on the specific form of meditation (e.g., open awareness or focused attention). The groups were matched on age, gender, socioeconomic status (education and income levels), depressive symptoms (Beck Depression Inventory; Beckham and Leber, 1985) and anxiety symptoms (Beck Anxiety Inventory; Beck and Steer, 1993; **Table 2**).

All subjects had normal or corrected-to-normal vision, and none had a history of neurological or psychiatric disorders, and no current use of psychoactive medications. All procedures were conducted in accordance with the Institutional Review Board at Baylor College of Medicine.

**Table 2:** Summary of demographic and behavioral variables.

	Controls (*n* = 30)	Meditators (*n* = 28)
Mean age	32.4 (9.3)	33.7 (11.2)
Female:Male ratio	14:16	13:15
Meditation experience	–	10.3 (8.1)
Education (years)	14.9 (1.6)	14.7 (1.8)
Income	2.6 (1.4)	2.7 (1.2)
BDI	4.8 (5.7)	2.8 (3.9)
BAI	4.2 (4.7)	3.1 (4.1)
MAAS	63.5 (13)	75.8 (9.8)*

Mean demographic variables were compared using two-sample *t*-tests assuming unequal variance. SD in parentheses. *Indicate significant differences (*p* < 0.05) between controls and meditators. BDI, Beck Depression Inventory; BAI, Beck Anxiety Inventory; MAAS, Mindful Attention Awareness Scale. Yearly income levels were coded as 1 = < $20K; 2 = 20–35K; 3 = 35–50K; 4 = 50–75K; 5 = 75–100K; and 6 = > 100K.

### EXPERIMENTAL PROCEDURES

The classical conditioning task had four scanning runs. The sequence in run 1 and 2 consisted of a yellow light cue of 1 s duration which was followed by juice delivery 6 s later (normal events). The time between individual pairings was randomly selected from between 4 and 14 s (at 2 s increments). In run 1 there were 23 such events and in run 2 there were 22 events. In the subsequent runs 3 and 4 there were 18 events in each run of which 6 events were catch events. For these catch events, the time from light cue to juice delivery was increased to 10 s. The light cues were presented and responses collected using NEMO (Human Neuroimaging Lab, Baylor College of Medicine). The stimuli were back-projected via an LCD projector onto a transparent screen positioned over the subjects’ head and viewed through a tilted mirror fixed to the head coil. Juice delivery was accomplished using a computer-controlled syringe pump (Harvard Apparatus, Holliston, MA, USA). Juice delivery consisted of 0.8 ml juice per event. Post-scanning, subjects reported enjoying the taste of the juice.

### FMRI DATA ACQUISITION

The anatomical and functional imaging was performed using 3 Tesla Siemens Trio scanners. High-resolution T1 weighted scans were acquired using an MPRAGE sequence (Siemens). The first five scans were discarded to allow for T1 equilibration effects. Functional imaging used an EPI sequence with a repetition time (TR) of 2000 ms, echo time (TE) = 25 ms, flip angle = 90°, 220 mm field of view (FOV), 64 × 64 matrix. Functional slices were oriented 30° superior-caudal to the plane through the anterior and posterior commissures in order to reduce signal drop-out due to magnetic field in-homogeneities (Deichmann et al., 2003). Each functional image was acquired in an interleaved way, comprising 37.4 mm axial slices for measurement of the BOLD effect (Ogawa et al., 1990), yielding 3.4 mm × 3.4 mm × 4.0 mm voxels.

### fMRI DATA ANALYSIS

Image pre-processing and data analysis was performed using SPM8 (Wellcome Department of Imaging Neuroscience, London, UK). Motion correction to the first functional scan was performed using a 6 parameter rigid-body transformation (Friston et al., 1996). The average of the motion-corrected images was co-registered to each individuals structural MRI using a 12 parameter affine transformation. Slice timing artifact was corrected, after which images were spatially normalized to the Montreal Neurological Institute (MNI) template provided in SPM8. Images were then spatially filtered with an 8 mm isotropic Gaussian kernel and for the analysis a high pass filter with a cut-off frequency at 1/128 Hz was applied. Following pre-processing a general linear model (GLM) was applied to the fMRI time-series where each event was modeled as single impulse response functions at light cue onset and at juice delivery onset (for runs 1 and 2). For runs 3 and 4 the model included the light cue, juice delivery during normal events, juice delivery during catch events, the absence of juice delivery at 6 s during catch events, and the absence of juice delivery during normal events (10 s after light cue). The model was convolved with the hemodynamic response function (HRF) including its temporal derivative to account for slight discrepancies in juice delivery time and duration. Residual effects of head motion were corrected for by including the 6 estimated motion parameters for each subject as regressors of no interest. The mean images from the first level analysis were entered into a second-level, random effects (RFX) analysis accounting for the between subject variance. An ANOVA model using the beta-estimates of the regressors of interest was used. Equal variance was not assumed, and thus non-sphericity correction was applied (Glaser and Friston, 2004). Using *t*-contrasts allowed us to test for correlations of the fMRI BOLD signal and the parameters of interest. The resulting *t* maps were subsequently transformed to the unit normal *z*-distribution to create a statistical parametric map for each contrast. The statistical results given were based on a single-voxel *t*-statistic corresponding to *p* < 0.05 corrected for multiple comparisons (FDR-corrected). The coordinates of all activations are reported in MNI space. SVC were applied in the *a priori* region in the bilateral putamen, where coordinates for the putamen was derived from McClure et al. (2003), specifically (-18, 4, 8) and (18 4 8), by applying 10 mm spheres around the peak MNI coordinates. Using the coordinates of the study cited above, we placed a 10 mm spherical ROI in the bilateral putamen and extracted beta estimates for normal and catch trials in each group for run 3 and run 4.

## Conflict of Interest Statement

The authors declare that the research was conducted in the absence of any commercial or financial relationships that could be construed as a potential conflict of interest.
